# A five-year trend analysis of malaria surveillance data in selected zones of Amhara region, Northwest Ethiopia

**DOI:** 10.1186/s12889-020-09273-5

**Published:** 2020-07-28

**Authors:** Damtie Lankir, Samrawit Solomon, Addisu Gize

**Affiliations:** 1 Department of Public Health, St. Paul’s Hospital Millennium Medical College, Addis Ababa, Ethiopia; 2 Department of Microbiology, St. Paul’s Hospital Millennium Medical College, Addis Ababa, Ethiopia

**Keywords:** Malaria surveillance, Data analysis, West Gondar zones, Northwest Ethiopia

## Abstract

**Background:**

Trend analysis of malaria surveillance data is essential to inform stakeholders on progress towards malaria control. From the total 387,096 cases of malaria reported in Amhara region in 2017, 167,079 (43.2%) cases were in Central, North and West Gondar zones. From this total figure of zones, 15,445 (9.2%) were ≤ 5 years which contributes 4% of cases in the region. So, the purpose of this study was to analyze trends of malaria parasite in Selected Zones of Amhara Region, Northwest Ethiopia.

**Methods:**

A Retrospective study was conducted on purposely selected Central, North and West Gondar zones from July 1–30/ 2018. Data were collected, entered, cleaned, analyzed and interpreted using Microsoft Excel-2010. Different tables, figures and maps were used to present results.

**Result:**

A total of 2,827,722 cases have been received a diagnostic test of; Microscopy 1,712,193(60.56%) and Rapid Diagnostic Test (RDT) 1,115,529(39.44%). Trends of total patients treated as confirmed and clinical malaria cases in July 2017–June 2018 were decreased to 139,297 (14%) as compared from July 2015–June 2016, 249,571(25%). From total cases received diagnostic tests only 1,003,391 (36%) were confirmed and clinical cases treated with antimalaria. Of these *Plasmodium falciparum and vivax* malaria cases were confirmed to be 1002,946 (99.96%) and clinical malaria cases were 445(0.04%), respectively.

**Conclusion:**

Risk of infection and diagnostic effort were high in West Gondar Zone. The Amhara public health institute including health Bureau, stakeholders and all responsible bodies should give special standing to highest malaria districts of West Gondar zone.

## Background

Malaria is the most important parasitic and vector born disease caused by the four species (*Plasmodium vivax, Plasmodium falciparum, Plasmodium malariae, Plasmodium ovale*) in Africa [1] which is a life-threatening disease. In areas with high transmission of malaria, children under 5 are particularly susceptible to infection, illness and death; more than two thirds (70%) of all malaria deaths occur in this age group. The number of under-5 malaria deaths has declined from 440,000 in 2010 to 285,000 in 2016. However, malaria remains a major killer of children under five years old, taking the life of a child every two minutes [2].

There have been over 9 million cases of malaria in the East African nation since January 2016, according to the report by the United Nation humanitarian office. In Zambia 119,593 (7.7/1000 population) cases were recorded in the first 11 weeks of 2017 compared to 73,019 and 82,328 cases for the same periods in 2016 and 2015. Even though Malaria prevention and control interventions have recently undergone major scale-up in Africa, Malaria disease burden is reported in several countries including Ethiopia and other East African countries. There is complexity within countries, including large geographical variation incidence and differing upward or downward trends between indicators [3].

Ethiopia is one of the most malaria endemic countries in Africa [4]. The population is reaching 105,350,020 in July 2017. Sixty eight percent of the population is at risk of malaria [5]. Approximately 75% of the geographic regions have significant malaria transmission risk. Among the leading communicable disease in Ethiopia, malaria accounts for about 30% of the overall Disability Adjusted Life Years lost [4].

According to the objective of National Malaria Policies and Strategies of Ethiopia, Program for Alternative Technology in Health (PATH), Malaria Control and Evaluation Partnership in Africa (MACEPA), December 2015 report; by 2017 and beyond, 100% of suspected malaria cases are diagnosed using Rapid Diagnostic Tests (RDTs) or microscopy within 24 h of fever onset (4–6). From 2009 to 2013 a total estimate of 13 million confirmed and clinical cases of malaria were reported in Ethiopia.

Stronger malaria surveillance systems could be enabled in endemic regions, to prevent outbreaks and resurgences, to track progress, and to hold governments and the global malaria community accountable from the updated information [5]. The information also needed to prioritize the most affected areas by malaria in different years and months and also may give baseline information for further study. So, the purpose of this study was to describe a five years malaria trend of Central, North and West Gondar zones and its districts (woreda’s) in Amhara region, Northwest Ethiopia.

## Methods

### Study setting

The study was conducted on purposively selected Central, North and West Gondar zones of Amhara regional state. Amhara region is one of the low land malarious regions of Ethiopia. Amhara National Regional state is one of the Ethiopia Regions with total population of 23,442,192 in 2017 estimate. The region consists of three major geographical zones, highlands or “Dega” (beyond 2300 m above sea level), semi-highlands or “Woyna Dega” (1500 to 2300 m above sea level) and lowlands “Kolla” or hot climatic zones (below 1500 m above sea level) accounting 25%, 44%, and 31% respectively [6]. The regional state was made up of 13 administrative zones.

Central, North and West Gondar Zones (previous North Gondar zone) are under the thirteen zones. These zones are located at a Latitude of 12° 44′ 59.99“ N and Longitude of 37° 00’ 0.00” E with an elevation of 1422 m above sea level. It is bounded by bordered on the South by lake Tana, West Gojjam, Awi and the Benishangul-Gumuz Region, on the West by South Sudan, on the North by the Tigray Region, on the East by Wag Hemra and on the South East by South Gondar Zone. Central Gondar Zone cover thirteen woreda, North Gondar zone cover seven Woereda and West Gondar zone cover six woredas. The first Health center in Ethiopia Kolla Diba is found in central Gondar zone. The total populations residing in Central Gondar were 2,896,928, North Gondar was 912,112 and West Gondar was 328,006. Generally a total of 4,137,046 populations were lived in these three zones. Of these Males were 2,107,981 and Female were 2,029,064 with a ratio of 1:1.

### Study design and period

A retrospective study design was conducted in Central, North and West Gondar zones form weekly reported malaria surveillance data of Amhara Public Health Institute (APHI), Public Health Emergency Management (PHEM) directorate from July 1–30/ 2018.

All Outpatient Department (OPD) and Inpatient Department (IPD) confirmed and clinical reported malaria cases residing in all districts/woredas of Central, North and West Gondar zones which covers 5 years period.

### Data collection instrument

Secondary data collection checklist was prepared and used to extract the documented malaria surveillance data of weekly reported cases from PHEM directorate office. This checklist contain variables like: Zone, Woreda, total population, Budget Year, Month, Reporting date, Total numbers in Outpatient department (OPD), Total incidence of parasite (IP), Total number of death, Blood Film (BF) test, Rapid Diagnostic Test (RDT), Total number of malaria (confirmed and clinical) in OPD, and age groups, etc.

### Inclusion and exclusion criteria

Since it was retrospectively study, a total of five year period confirmed and clinically treated cases for malaria were included in the study and all incomplete data were excluded.

### Data collection process

A regional weekly collected malaria data base was extracted to zones and woredas through filtering of important variables and filtered data were used for analysis. Total confirmed and clinically treated cases were cheeked as total inpatient and outpatient visited cases to have a quality data.

### Data processing and analysis

Data were entered, processed and analyzed into Microsoft office Excel 2010. A descriptive analysis, using mean and percentage was calculated. Different graphs and tables were used to present trends of malaria cases and total population.

### Ethical consideration

Permission letter was gained from Amhara public health institute; research and technology transfer directorate; and permission letter was secured from Amhara Public Health Institute (APHI) stated the ethics number as (APHI/ 5011, 06/02/11). The purposes and the importance of the study were stated this unique objective of the study for the region which serve as base line information to Public Health Emergency Management (PHEM) directorate office, and for different stake holders. PHEM directorate is the owner of this five years data and it direct stakeholder or programmer for malaria prevention and intervention measures. Confidentiality was assured at all levels of the study using password protected computer and through deleting all identifiers.

### Operational definition

Suspected malaria case: - Clinical diagnosis of malaria is made in a patient who has fever or history of fever in the last 48 h and lives in malaria-endemic areas or has a history of travel within the last 30 days to malaria-endemic areas [7].

Confirmed malaria case: - A suspected case of malaria in which malaria parasites have been verified by microscopy or RDT.

Annual blood examination rate: - Smears examined in a year X 100 / Total population.

Annual falciparum incidence: - Total positive PF in a year × 1000 / Total population.

Annual parasite incidence: - Total number of positive slides for malaria parasite in a year × 1000 / Total population.

## Results

### Trends of malaria cases by zones and years

**Fig. 1:**
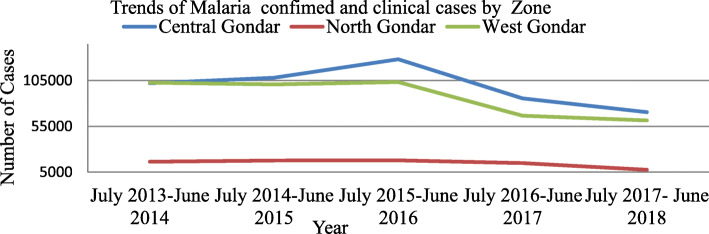
Five years trends of total confirmed and clinical malaria Cases in Central, North and West Gondar Zones from July 2013–June 2018

Within five years from July 2013–June 2018 a total of 5,735,065 populations were attended health facilities in Central, North and west Gondar zones. The average annual health facility attendance rate of these zone were a total of 1,147,013 patients (285/1000 population) each year. In five years period a total of 2,827,722 cases were receiving a diagnostic test of Microscopy 1,712,193 (60.56%) and RDT 1115529 (39.44%). From total cases received diagnostic tests only 1,003,391 (36%) were confirmed and clinical cases treated with antimalaria. Of these treated with antimalarial cases, *Plasmodium falciparum and vivax* malaria cases were 1002,946 (99.96%) and clinical malaria cases were 445(0.04%) in Central, North and West Gondar zones. Among the total confirmed malaria cases 8623 (0.86%) were pregnant women positive for malaria. Average annual malaria cases were 200,589 (55 cases per 1000 population). The trends of total clinically and confirmed malaria cases were increased from 220,406(22%) in July 2013–June 2014 to 249,474 (25%) in July 2015–June 2016 in Central, North and West Gondar zone. In July 2017–June 2018 total patients treated as confirmed malaria cases were decreased by 139,296 (14%) as compared to July 2015–June 2016; 249,474(25%). Trends of malaria cases by zones were presented in Fig. 1.

### Trends of confirmed malaria species by years and zones

**Fig. 2:**
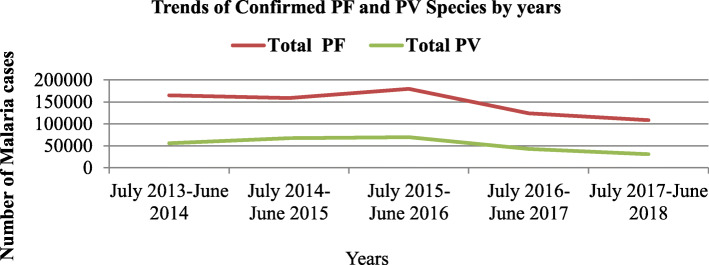
Trends of PF and PV cases in Central, North and West Gondar zones from July 2013–June 2018

From a total of 1002, 946 confirmed malaria cases in Central, North and West Gondar zones; *Plasmodium falciparum* species were 736,149 (73.4%) and *Plasmodium vivax* were 266,797 (26.6%). Within five years period in West, Central and North Gondar Zones *Plasmodium falciparum* accounts 337,807(77.7%), 351,433(71.1%) and 46,909(63.2%), respectively. *Plasmodium vivax* infection were 96,836(22.3%) in West Gondar, 142,646(28.9%) in Central Gondar and 27,315(36.8%) in North Gondar zones. On average the number of malaria cases per year were 86,929(273/1000 population), 98,816 (35/1000 population) and 14,845(17/1000 population) in West, Central and North Gondar zones respectively. Total confirmed PF and PV species by years were presented in Fig. 2.

### Trends of malaria cases by months and epidemiological (EPI) weeks

**Fig. 3:**
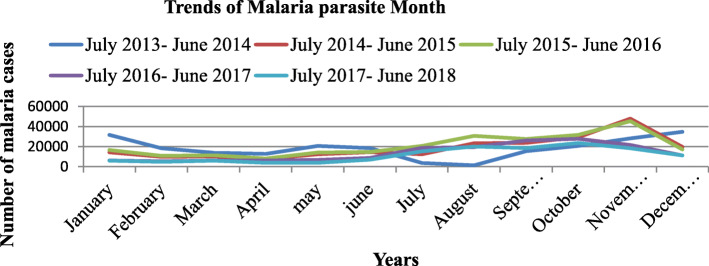
Transmition of malaria parasite by month in Centeral, North and West Gondar Zones from July 2013–June 2018

**Fig. 4:**
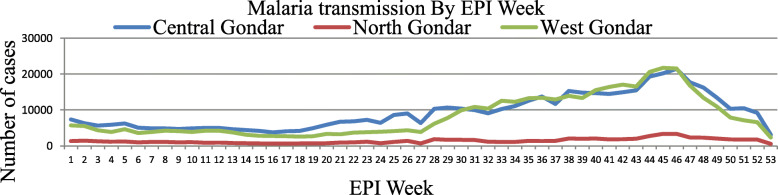
Malaria transmission by EPI weeks in Centeral, North and West Gondar Zones from July 2013–June 2018

The trends of malaria cases in July 2014–June 2015 and July 2016–June 2017 were high on November and lowest on April in both years. In July 2017–June 2018 highest number of cases was registered on October and lowest numbers of cases were reported on April. The highest malaria cases were reported in EPI week of 45, 46 and 47 in these three zones within five years period. Trends of malaria transmission by months and EPI week were presented in Figs. 3 and 4 respectively.

### Trends of PF and PV transmission by months

**Fig. 5:**
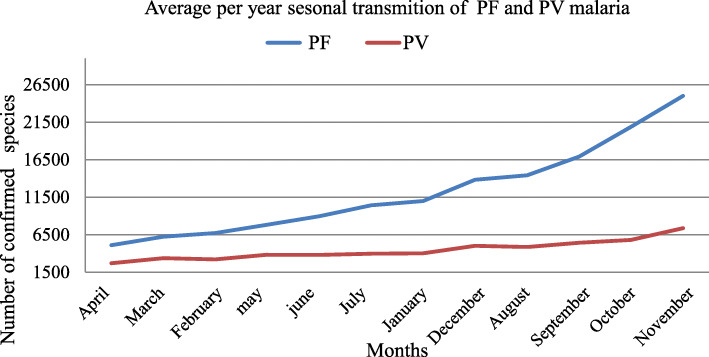
Monthly confirmed malaria cases average distribution over five years by *Plasmodium falciparum and vivax* Species in Central, North and west Gondar zone, Ethiopia, July 2013–June 2018

Annual average highest monthly *Plasmodium falciparum* transmission were observed in November with 24,993(17%) cases followed by October 20,873(14%) and September 16,892(11%) cases respectively. *Plasmodium vivax* transmission were also high in November, October and September with 7365(14%), 5813(11%) and 5431(10%) cases respectively at Central, North and West Gondar zones. Average Seasonal transmissions of falciparum and vivax malaria by months were presented in Fig. 5.

### Malaria morbidity and mortality

**Fig. 6:**
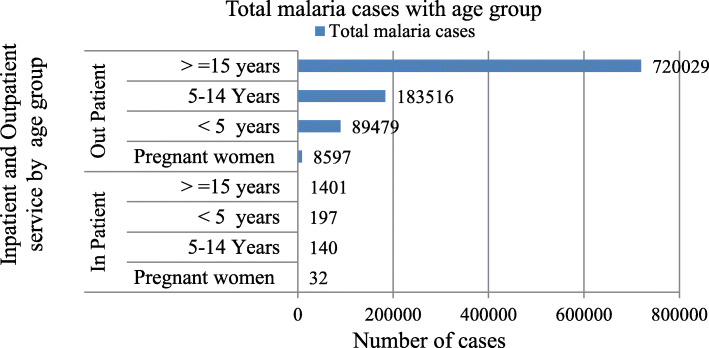
Number of outpatient and inpatient clinical and confirmed malaria cases by age group in Central, North and West Gondar Zones from July 2013–June 2018

**Fig. 7:**
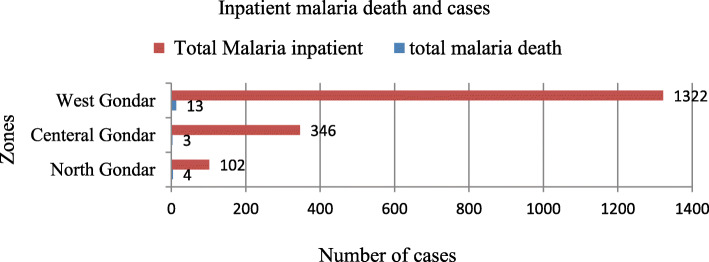
Inpatient malaria cases and inpatients to all disease in Central, North and West Gondar zones from July 2013–June 2018

Morbidity due to malaria was reported in all age groups including pregnant mothers. Within five years period > 15 years of age were more affected by malaria. Out of 1,003,391 total confirmed and clinical malaria cases obtaining outpatient and inpatient service, > 15 years of age accounts 721,430 (71.89%) followed by 5–14 years of age 183,656 (18.31%), < 5 years 89,676 (8.94%) and pregnant women 8629 (0.86%). All malaria cases treated in inpatient and OPD by age group were presented in Fig. 6. Within five years period a total of 1770 (0.18%) cases received inpatient malaria service. Average annual inpatient malaria cases in West Gondar zone were 265 (83 per 100,000 population) followed by Central Gondar 69 (three per 100,000 populations) and North Gondar 20 (two per 100,000 populations). There were a total of 20 deaths in five years period. Case Fatality Rate (CFR) in North Gondar Zone were three (4/100,000 population), West Gondar zone were 13(3/100,000 population) and Central Gondar zone were four (0.89/100,000 population). In patient of malaria case and inpatient of all disease by zone were presented in Fig. 7.

### *Plasmodium falciparum and vivax* species transmission

**Fig. 8:**
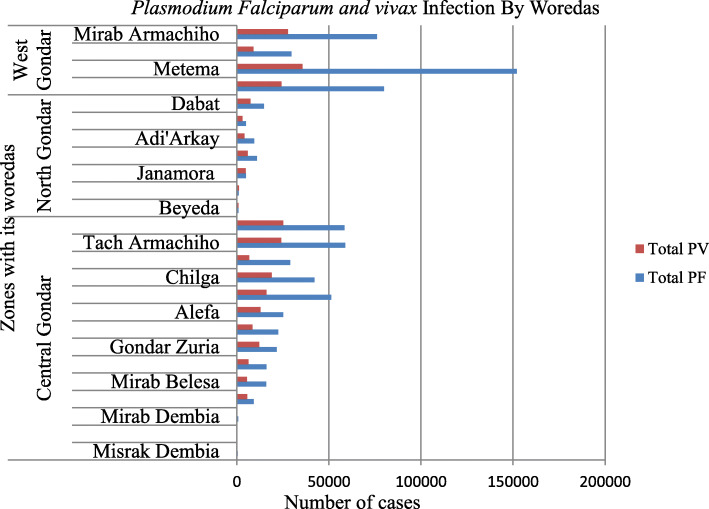
Distribution of *Plasmodium falciparum and vivax* cases of districts from July 2013–June 2018

*Plasmodium falciparum* cases in West Gondar Zone of; Matema were 152,017 (45%), Quara were 79,894(24%) and Mirab Armachio were 71,137(23%) within five years period compared to other woredas in the zone. *Plasmodium vivax* in Metema were 35,649(37%) followed by Mirab Armachio 27,843(29%) from west Gondar zone and Tegede Woreda 25,220 (18%) from central Gondar zone. Transmissions of PF and PV in Central, North and West Gondar zone by its woredas were presented in Fig. 8.

### Annual parasite incidence (API) by zones and districts (woredas)

**Fig. 9:**
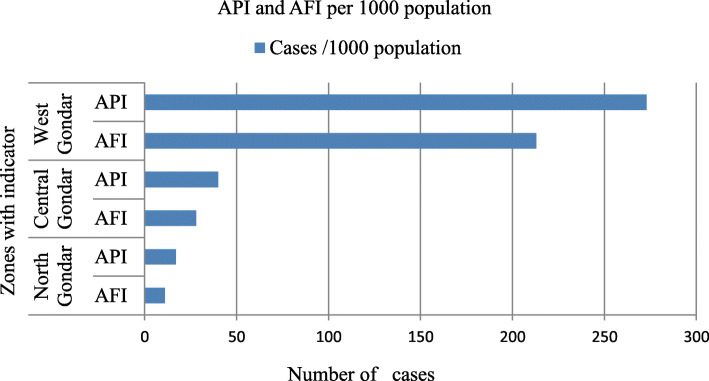
API of malaria in Central, North and West Gondar zones from July 2013–June 2018

Annual parasite incidence of malaria case in West Gondar zone were 86,929 (273/1000 population), Central Gondar zone were 98,816 (40/1000 population) and North Gondar zone were 14,845 (17/1000 population). The annual parasite incidence of Mirab Armachio, Gendawuha, Metema and Quara woreda were 20,796 (481/1000 population), 7754 (298/1000 population), 37,553 (269/1000 population) and 20,846(191/1000 population) cases respectively from West Gondar Zone. Annual parasite incidences by zones were presented in Fig. 9.

### Annual blood examination and test positivity rates

**Fig. 10:**
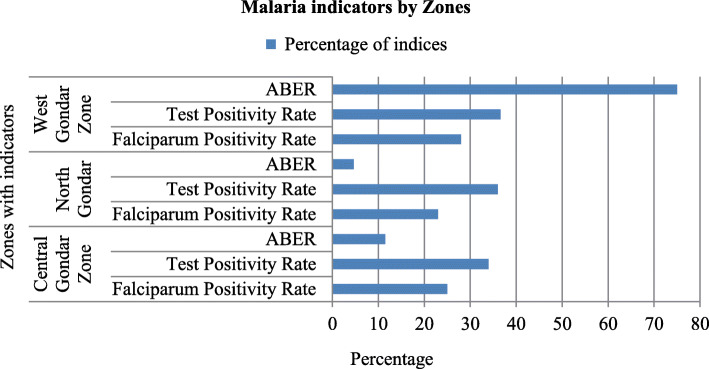
Average ABER and test positivity rate in Central, North and West Gondar zones from July 2013–June 2018

The Annual blood examination rate (ABER) of West Gondar zone was 237,675 (75%) followed by Central Gondar 286,527 (12%) and North Gondar zone 41,342 (5%). Test positivity rate of was greater in West Gondar zone. ABER test and falciparum positivity rate of zone were presented in Fig. 10.

## Discussion

The overall five years Malaria trend analysis of Central, North and West Gondar zones indicates that the incidence of confirmed outpatient and inpatient malaria cases were increased from 220,406 (22%) in July 2013–June 2014 to 249,474 (25%) in July 2015–June 2016 and decreased to 139,296 (14%) in July 2017–June 2018. Average annual malaria cases of 200,589 (55 cases per 1000 population) were identified and treated with anti-malaria in Central, North and west Gondar zones which contributes 2.23% of East Africa nations malaria cases. These results shows that there were an increment of cases compared to Namibia 24,682 (12.33/1000 population) cases in 2016, Zambia 119,593 (7.7/1000 population) and highly affected regions of Botswana 794(0.36/1000 population) malaria cases 2017 WHO report [3, 8]. This variation may be due to strong commitment of malaria controlling strategies of the countries, geographical variation and accessibility of controlling materials.

The number of malaria death were also decreased from 14 (0.34/100,000 Population) in July 2013–June 2014 to three (0.07/100,000 Population) in July 2017–June 2018 in the central, North and West Gondar zones. This result shows a different report in Burundi 4000(4/10,000 population) cases killed due to malaria and similar to five (0.23/100,000 Population) deaths in Botswana 2017 WHO report [3, 5, 8]. This significant difference may be due to outbreak conditions of the country and not timely treatment or arrival of cases to treatment centers and it requires further investigations.

A total of 1770 (0.18%) cases received inpatient malaria service within five years period. Average annual inpatient malaria cases in West Gondar zone were high 265 (83 per 100,000 population) as compared to Central Gondar 69 (three per 100,000 populations) and North Gondar 20 (two per 100,000 populations). From 1770 inpatient cases only 20 deaths were recorded in five years period in these zones. Case Fatality Rate (CFR) in West Gondar zone were 13(3/100,000 population), which is high as compared to Central Gondar zone four (0.89/100,000 population). This zonal inpatient death variation may be due to high 265 (83/100,000 population) inpatient cases in related to central Gondar Zone 69(3/100,000 Population) inpatient cases and not severely arrival of cases to the health institutions and continuous follow up cases in the inpatient service.

Within five years period > 15 years of age were more affected by malaria. Out of 103,391 total confirmed and clinical malaria cases obtaining outpatient and inpatient service, > 15 years of age accounts 720,029(71.76%) followed by 5–14 years of age 183,516(18.29%) in outpatient visit. Within five years period in Central, North and West Gondar zones inpatient malaria cases were 1770 (17.64/10,000 population) with a discharge rate of 1750 (98.87%). Next to > 15 years of age 1401(14/10,000 population), < 5 years of age 197(2/10,000 population) were more affected compared to 5–14 years of age 140(1.4/10,000 population) in inpatient service. This study shows slightly similar to studies conducted in other part of Ethiopia in 2015 and in North Shoa, Ethiopia between 2013 and 2017 [9]. This may be due to geographical similarity of reported zones and woredas and similarity of study design.

Highest monthly *Plasmodium falciparum* transmission were observed in November with 249, 93(17%) cases followed by October 20,873(14%) and September 16,892(11%) cases respectively. *Plasmodium vivax* transmission were also high in November, October and September with 7365(14%), 5813(11%) and 5431(10%) cases respectively at Central, North and West Gondar zones. This result showed that a different study conducted in Sibu Sire District, East wolega zone, with a highest peak in June 18.9% followed by May, November, and July with prevalence rate, 13.3, 13.2, and 11.2%, respectively. The prevalence rate in October, August, and September were 9.4, 8.7, and 7%, respectively [10]. The variation may be due to amount of rain fall, climatic variation, geographical difference and year of study.

From a total of 1002, 946 confirmed malaria cases in Central, North and West Gondar zones; *Plasmodium falciparum* species were 736,149 (73.4%) and *Plasmodium vivax* were 266,797 (26.6%). This result shows difference in the study conducted in South Wollo zone Kombolcha health center from January to December 2016, *Plasmodium falciparum* accounted for 1243 (60.2%) while *P. vivax* accounted for 734 (35.5%) cases [11]. In this study PF in West and Central Gondar zone was the most frequently reported species. The reason for this variation is not clear; however, may be due to the previous study focus on health center and the current study cover zonal level and may need further study.

API and ABER of West Gondar zone were High 86,929(273/1000 population) and 237,675 (75%) respectively compared to Central Gondar zone of API 98,816 (40/1000 population) and ABER 286,527(12%). Annual parasite incidence were high in Mirab Armachiho, Genda wuha and Metema woreda 20,796(481/1000 population), 7753 (298/1000 population) and 37,553 (269/1000 population) respectively compared to central and North Gondar zones of its woredas. Annual blood examination rate of Gendawuha 35,983(138%) were high compared to Mirab Armachiho 285,743 (132%) and Metema woreda 90,723(65%). This result shows that rate of infection with transmission and diagnostic efforts were high in West Gondar zone of all woredas. This high risk may be due to geographical location of the woredas and increased the number of daily labors to investment area.

Annual Falciparum incidence were high in West Gondar zone 67,561 (213/ 1000 population) compared to central Gondar Zone 70,287 (28/1000 population) and North Gondar 9382 (11/1000 population). Tsegede 11,770 (135/1000 population) and Tacharmachiho Woreda 111,770 (110/1000 population) were have high annual falciparum incidence relative to other central Gondar zone Woredas. From west Gondar zone; Mirabaremachio 15,222(352/1000 population), Gendawuha 35,985(229/1000 population) and Metema 90,723(218/1000 population) were the leading woreda by Annual falciparum incidence. This result shows that a similar report to 167,079 (43.2%) cases of previous North Gondar Zone 2017 Health Bureau PHEM report [7]. This difference may be due to study period and study design variation and it also need further investigation.

### Limitations of the study

*Plasmodium falciparum* and *vivax* infection by pregnancy status, age categories and death of malaria with species infection were not analayzed. Distrbution of malaria by investement areas with its population at risk were not identified. Additionally, any malaria intervention activities that had been taken to control malaria were not collected from the study area.

## Conclusion

Trends of total clinical and confirmed malaria cases were decreased from year to year. Rate of infection and diagnostic effort were high in West Gondar Zone. Malaria transmission were high in Mirabaremachiho 15,222 (352/1000 population), Gendawuha 35,985 (229/1000 population), Metema 90,723 (218/1000 population) from West Gondar zone and Tsegede 11,770 (135/1000 population) followed by Tacharmachiho 111,770 (110/1000 population) woreda from central Gondar zone. Amhra Public Health Institute and Regional Health Bureau should give special standing to highest annual parasite incidence area of west Gondar zone and highly transmitted districts/ woredas specially Mirabarmachio, Gendawuha and Metema woredas.

## Data Availability

The authors confirm that all data underlying the findings are fully available without restriction. All relevant data are within the manuscript.

## Abbreviations

ABER: Annual Blood Examination Rate
AFI: Annual Falciparum Incidence
APHI: Amhara Public Health Institute
API: Annual Parasite Incidence
CFR: Case Fatality Rate
EPI: Epidemiology
IPD: In Patient Department
MACEPA: Malaria Control and Evaluation Partnership in Africa
OPD: Out Patient Department
PATH: Program for Alternative Technology in Health
PF: *Plasmodium falciparum*
PHD: Public Health Department
PHEM: Public Health Emergency Management
PV: *Plasmodium vivax*
RDT: Rapid Diagnostic Test
SPHMMC: St. Paul’s Hospital Millennium Medical College
UN: United Nation
WHO: World Health Organization
